# Acute effect of antiseizure drugs on background oscillations in *Scn1a* ^A1783V^ Dravet syndrome mouse model

**DOI:** 10.3389/fphar.2023.1118216

**Published:** 2023-03-20

**Authors:** Shir Quinn, Marina Brusel, Mor Ovadia, Moran Rubinstein

**Affiliations:** ^1^ Goldschleger Eye Research Institute, Sackler Faculty of Medicine, Tel Aviv University, Tel Aviv, Israel; ^2^ Department of Human Molecular Genetics and Biochemistry, Sackler Faculty of Medicine, Tel Aviv University, Tel Aviv, Israel; ^3^ Sagol School of Neuroscience, Tel Aviv University, Tel Aviv, Israel

**Keywords:** Dravet syndrome, background oscillations, mouse model, antiseizure, spectral modulation

## Abstract

Dravet syndrome (Dravet) is a rare and severe form of developmental epileptic encephalopathy. Antiseizure medications (ASMs) for Dravet patients include valproic acid (VA) or clobazam (CLB), with or without stiripentol (STP), while sodium channel blockers like carbamazepine (CBZ) or lamotrigine (LTG) are contraindicated. In addition to their effect on epileptic phenotypes, ASMs were shown to modify the properties of background neuronal activity. Nevertheless, little is known about these background properties alterations in Dravet. Here, utilizing Dravet mice (DS, *Scn1a* ^A1783V/WT^), we tested the acute effect of several ASMs on background electrocorticography (ECoG) activity and frequency of interictal spikes. Compared to wild-type mice, background ECoG activity in DS mice had lower power and reduced phase coherence, which was not corrected by any of the tested ASMs. However, acute administration of Dravet-recommended drugs, VA, CLB, or a combination of CLB + STP, caused, in most mice, a reduction in the frequency of interictal spikes, alongside an increase in the relative contribution of the beta frequency band. Conversely, CBZ and LTG increased the frequency of interictal spikes, with no effect on background spectral properties. Moreover, we uncovered a correlation between the reduction in interictal spike frequency, the drug-induced effect on the power of background activity, and a spectral shift toward higher frequency bands. Together, these data provide a comprehensive analysis of the effect of selected ASMs on the properties of background neuronal oscillations, and highlight a possible correlation between their effect on epilepsy and background activity.

## Introduction

Dravet syndrome (Dravet) is a rare and severe form of developmental epileptic encephalopathy (DEE). Most cases are caused by heterozygous *de novo* mutations in the *SCN1A* gene, encoding the alpha subunit of the voltage-gated sodium channel type I (Na_V_1.1). The first sign of the disease is febrile seizures that soon progress to refractory spontaneous seizures with developmental delays (Dravet, 2011; Gataullina and Dulac, 2017). Electroencephalogram (EEG) background activity is typically normal at the onset of seizures, but interictal epileptic discharges can be observed (Holmes et al., 2012; Minato and Myers, 2021; Wirrell et al., 2022).

Dravet seizures are difficult to control, even with polytherapy. Drug treatment includes valproic acid (VA) or clobazam (CLB) as first-line drugs, with stiripentol (STP), fenfluramine, or cannabidiol as a second-line add-on treatment. Conversely, sodium channel blockers such as carbamazepine (CBZ) or lamotrigine (LTG) are contraindicated, as these can aggravate the seizures (Wirrell et al., 2022).

Dravet mouse (DS mice) models are an exceptionally good genocopy and phenocopy of the human syndrome. DS mice are mostly asymptomatic until their fourth week of life (postnatal day (P) 20–27) when they begin exhibiting spontaneous seizures and profound premature mortality (Yu et al., 2006; Ogiwara et al., 2007; Miller et al., 2014; Mantegazza and Broccoli, 2019; Ricobaraza et al., 2019). Electrocorticography (ECoG) recordings from DS mice in their fourth week of life demonstrated an unaltered spectral profile, similar to Dravet patients (De Stasi et al., 2016; Fadila et al., 2020; Gerbatin et al., 2022). However, analyses of the non-normalized power spectral density (PSD) in naïve, unmedicated mice showed reduced power. Moreover, mice that died prematurely had the lowest power (Fadila et al., 2020; Gerbatin et al., 2022).

The effect of ASMs on background EEG activity was studied before, but not in Dravet. VA was shown to reduce the power of background EEG in patients with idiopathic generalized epilepsy, and juvenile myoclonic epilepsy (Clemens et al., 2006; Moon et al., 2022), and CLB caused spectral changes in rabbits (Gerhards, 1978) and kindled rats (Harris et al., 1988). Here, utilizing DS mice (*Scn1a* ^A1783V/WT^) we show that acute administration of Dravet-recommended drugs, VA, CLB, a combination of CLB + STP, or STP alone, was associated with a reduction in the frequency of interictal spikes in most of the tested mice, along with a significant increase in relative beta band contribution to the total power.

Conversely, CBZ and LTG, contraindicated in Dravet, increased the frequency of interictal spikes with no effect on the spectral properties of background activity. Moreover, we uncovered a correlation between the reduction in interictal spike frequency and the redistribution of power towards increased beta and gamma band contribution. Together, these data demonstrate that ASMs modulate background neuronal activity and that these modulations correlate with their effect on seizure control.

## Methods

### Animals and surgery

All animal experiments were approved by the Institutional Animal Care and Use Committee (IACUC) of Tel Aviv University. Mice used in this study were housed in a standard animal facility at the Goldschleger Eye Institute at a constant (22°C) temperature, on 12-h light/dark cycles, with *ad libitum* access to food and water.

DS mice harboring the global *Scn1a* ^A1783V/WT^ mutation on pure C57BL/6J background were generated as described before (Fadila et al., 2020; Almog et al., 2021), by crossing conditional *Scn1a* ^A1783V^ males (The Jackson Laboratory, stock #026133) with CMV-Cre females (The Jackson Laboratory, stock #006054).

Electrode implantation was done on P19-P25, as previously described (Fadila et al., 2020). Briefly, the mice were anesthetized (ketamine/xylazine, 191/4.25 mg/kg), and carprofen (5 mg/kg) was given for analgesia prior to surgery and 24 h post-op. A midline incision was made above the skull, and fine silver wire electrodes (130 μm diameter bare; 180 μm diameter coated) were placed at visually identified locations: bilaterally above the somatosensory cortex; a reference electrode was placed on the cerebellum midline; ground and electromyography (EMG) electrodes were placed subcutaneously over the left and right shoulders, respectively. The electrodes were secured using dental cement and connected to a micro-connector system. Mice were allowed to recover for at least 48 h before recording.

### Drugs administration

The drugs that were used were: valproic acid (VA, 300 mg/kg in saline; Sigma-Aldrich), clobazam (CLB, 10 mg/kg in sesame oil; EDQM), stiripentol (STP, 150 mg/kg in sesame oil; Angene Chemical), a combination of clobazam and stiripentol (CLB + STP, 5 mg/kg + 100 mg/kg in sesame oil), carbamazepine (CBZ, 20 mg/kg in 30% polyethylene glycol 400; Alomone Labs) and lamotrigine (LTG, 10 mg/kg in 30% polyethylene glycol 400; Alomone Labs). Drug dosages were chosen to reach therapeutic-relevant concentrations following acute administration (Hawkins et al., 2017). All drugs were administered as a single IP injection in a volume of 10 ml/kg.

### Data acquisition and ECoG signal pre-processing

Video-ECoG recordings were obtained from freely behaving mice at P21-P27, as described before (Fadila et al., 2020). The recordings lasted for 4 h, between 10 a.m. and 5 p.m. After 2 hours of recording, one of the drugs was administered. The first 30 min of the recording and the first 30 min post-drug administration were considered acclimation periods and were not taken for analysis. The EMG signal was denoised and smoothed using a custom-written Python script, based on methods described before (Solnik et al., 2010). Briefly, the raw EMG signal was denoised with the Teager-Kaiser Energy Operator (TKEO), and the output was rectified and smoothed across a 3 Hz cut-off low pass filter. Next, to calculate spectral properties, the recording was divided into 5 s epochs with a 2.5 s sliding window. Power spectral densities of the ECoG were computed for each epoch using Welch’s method with a 50% overlap Hann window. The power bands were defined as: delta, 0.9–3.9 Hz; theta, 4.8–7.8 Hz; alpha, 8.7–11.7 Hz; beta 12.6–29.2 Hz; gamma, 30.2–99.6 Hz. We used a custom-written threshold algorithm in python to extract artifact-free epochs where the mice were awake and not moving. An epoch was taken for analysis if all conditions were met: *i*) the delta power of the epoch was lower than the median delta power; *ii*) the theta to delta ratio was lower than the 75% quantile of the entire 1.5 h recording block; *iii*) the enveloped EMG was less than two times the harmonic mean throughout the entire epoch (Weber et al., 2015; Barger et al., 2019). Finally, all automatically selected epochs were concatenated and manually inspected. For each mouse, at least 100 epochs were analyzed. Then, the data were averaged across the different epochs. Thus, only one data point before drug administration, and one data point after ASM administration, was considered for statistical analysis. To quantify phase synchrony between the left and right somatosensory electrodes, we calculated the interhemispheric coherence coefficient (coherence) using a custom-written Python script, based on the coherence coefficient equation as described before (Bastos and Schoffelen, 2016).

Custom-written codes are available at https://github.com/shirquinn/Rubinstein_Lab.git.

To quantify the frequency of interictal spikes, we applied a bandpass filter between 0.5 and 60 Hz and used the spike histogram module in LabChart 8 software (ADInstruments). The threshold was set to 4–5 times the standard deviation of the filtered signal, with a 50 ms pretrigger interval and 250 ms maximal total duration. The spikes were then manually inspected in the scope view window, and artifacts were rejected.

### Thermally-induced seizures

Thermal induction of seizures was performed as previously described (Almog et al., 2021). Briefly, 30 min after injecting one of the selected drugs, the body temperature was increased by 0.5°C every 2 min using a heat lamp attached to a feedback temperature controller (TCAT-2DF, Physitemp Instruments), until a generalized tonic-clonic seizure (GTC) was provoked. Drugs that were used for assessing thermal induction (TI) of seizures were: *i*) VA (300 mg/kg); *ii*) CLB + STP (5 mg/kg + 100 mg/kg). Control DS mice were injected with the appropriate vehicle control.

### Statistics

Statistical analyses were performed using Prism 9 (GraphPad Software). Data are reported as mean ± SE. Statistical analysis utilized Student’s t-test or the non-parametric Mann-Whitney test (for data with normal distribution or data that did not distribute normally). For comparison between wild-type (WT) and DS mice and the effect of drugs on thermal-induced seizures, we used the Student’s t-test (for data with normal distribution) or the non-parametric Mann-Whitney test (for data that did not distribute normally). To compare WT or DS mice before and after drug administration, we used paired Student’s t-test (for data with normal distribution), or the Wilcoxon matched-pairs signed rank test (for data that did not distribute normally). To compare the effect on normalized power spectral density (PSD) or coherence, we used Two-Way repeated measures ANOVA followed by Sidak posthoc analysis. For the correlation analysis, the Spearman rank-order correlation coefficient was used. Statistical analysis of pie charts was performed using Fisher’s exact test. We considered *p* < 0.05 as statistically significant.

## Results

### Reduced background ECoG power and interhemispheric coherence in DS mice

**FIGURE 1:**
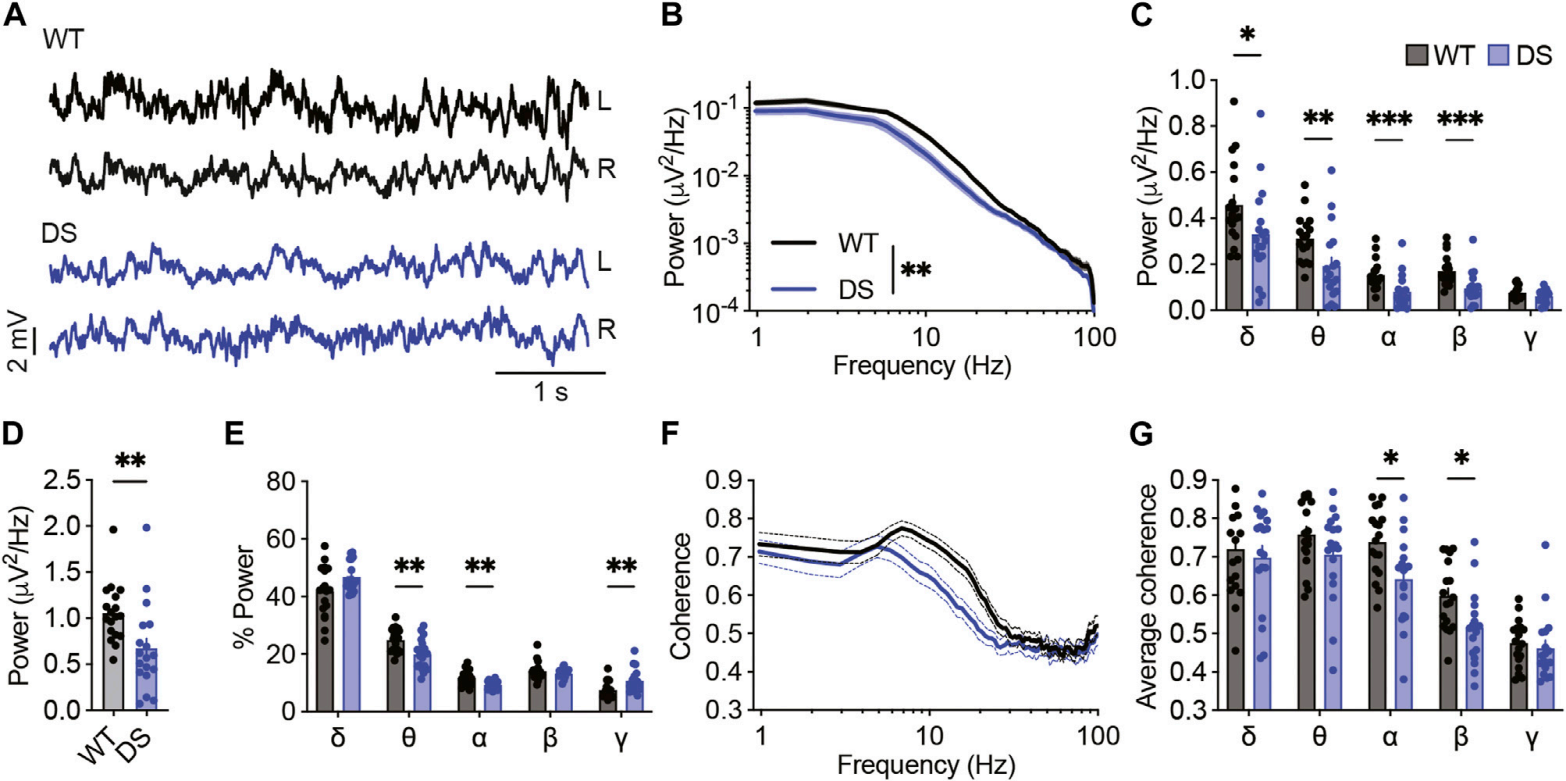
Reduced power of background activity and interhemispheric coherence in DS mice. **(A)** Representative traces of background ECoGs in WT and DS mice. **(B)** ECoG power density profiles. **(C)** Total power for the following frequency bands: δ, 0.9–3.9 Hz; θ, 4.8–7.8 Hz; α, 8.7–11.7 Hz; β, 12.6–29.2 Hz; γ, 30.2–99.6 Hz. **(D)** Total power. **(E)** The relative power (%) of each frequency band. **(F)** Interhemispheric coherence plotted over the 1–100 Hz spectrum. **(G)** The mean interhemispheric coherence for each frequency band. WT, n = 18; DS, n = 18. *p < 0.05, **p < 0.01, ***p < 0.001.

Global neuronal activity in juvenile WT and DS littermates (P21-P27) was examined using simultaneous video-ECoG recordings, followed by quantitative background activity spectral properties analyses. In accordance with previous reports (Fadila et al., 2020; Gerbatin et al., 2022), a global reduction in total power was seen in DS mice at this age, observed in all frequency bands up to 30 Hz (Figures 1A–D). Extracting the relative power for each band revealed a higher contribution of the gamma band and a reduced contribution of theta and alpha bands (Figure 1E).

While seizure activity is characterized by synchronized activity (Supplementary Figure S1), altered interhemispheric phase synchrony, or coherence, of background non-epileptic segments was found in various brain disorders, including epilepsy and autism (Bowyer, 2016). Here, we found reduced coherence in DS mice compared to WT controls, mainly in the alpha and beta frequency bands (Figures 1F, G), indicating deficits in synchrony between frequency components of background ECoG. Together, quantitative analyses of background ECoG recordings from DS mice demonstrated reduced power and coherence.

### Dravet-prescribed ASMs modulate background ECoG activity and spectral properties in DS mice

Thermally-induced seizures are a hallmark phenotype of Dravet, and multiple studies examined the effect of ASMs on the temperature threshold of these seizures (Hawkins et al., 2017; Pernici et al., 2021). However, this measurement provides a unidimensional description of a transition into a convulsive seizure and cannot be adopted for clinical use. We reasoned that analyses of the properties of background oscillations might provide additional insights into the effect of ASMs in Dravet. From these recordings, we also examined the effect of these ASMs on epilepsy, quantifying the change in the frequency of interictal epileptic spikes. Interictal spikes reflect synchronous neuronal firing. They are strongly associated with epilepsy and were shown to have adverse effects on brain development and contribute to cognitive impairment (Holmes, 2013). Although the relationship between interictal spikes and spontaneous seizures is not clear (Avoli et al., 2006), quantification of their frequency is commonly used to evaluate the severity of epilepsy and drug response in animal models. We focused on the acute effect of ASMs due to challenges with long-term ECoG recording and prolonged drug administration in immature mice.

Valproic acid (VA) is a broad-spectrum antiseizure medication and the drug of choice as a first-line treatment for Dravet. Specifically, VA was shown to reduce the frequency of spontaneous seizures in 50%–70% of patients (Shi et al., 2016; Hawkins et al., 2017). In DS mice, chronic treatment with VA protected from spontaneous convulsive seizures (Hawkins et al., 2017), but its acute effects on background ECoG activity were not assessed.

When given acutely, VA has several molecular targets: *i*) increasing GABA level by reducing its degradation *via* inhibition of GABA transaminase and succinic semi-aldehyde dehydrogenase; *ii*) potentiation of the activity of GABA_A_ receptors; *iii*) direct inhibition of voltage-gated sodium channels and T-type voltage-gated calcium channels; *iv*) inhibition of NMDAR. In addition, long-term VA administration, which was not tested here, results in histone deacetylase inhibition, positive modulation of M currents, and activation of the ERK and JNK pathways (Rosenberg, 2007; Zanatta et al., 2019).

**FIGURE 2:**
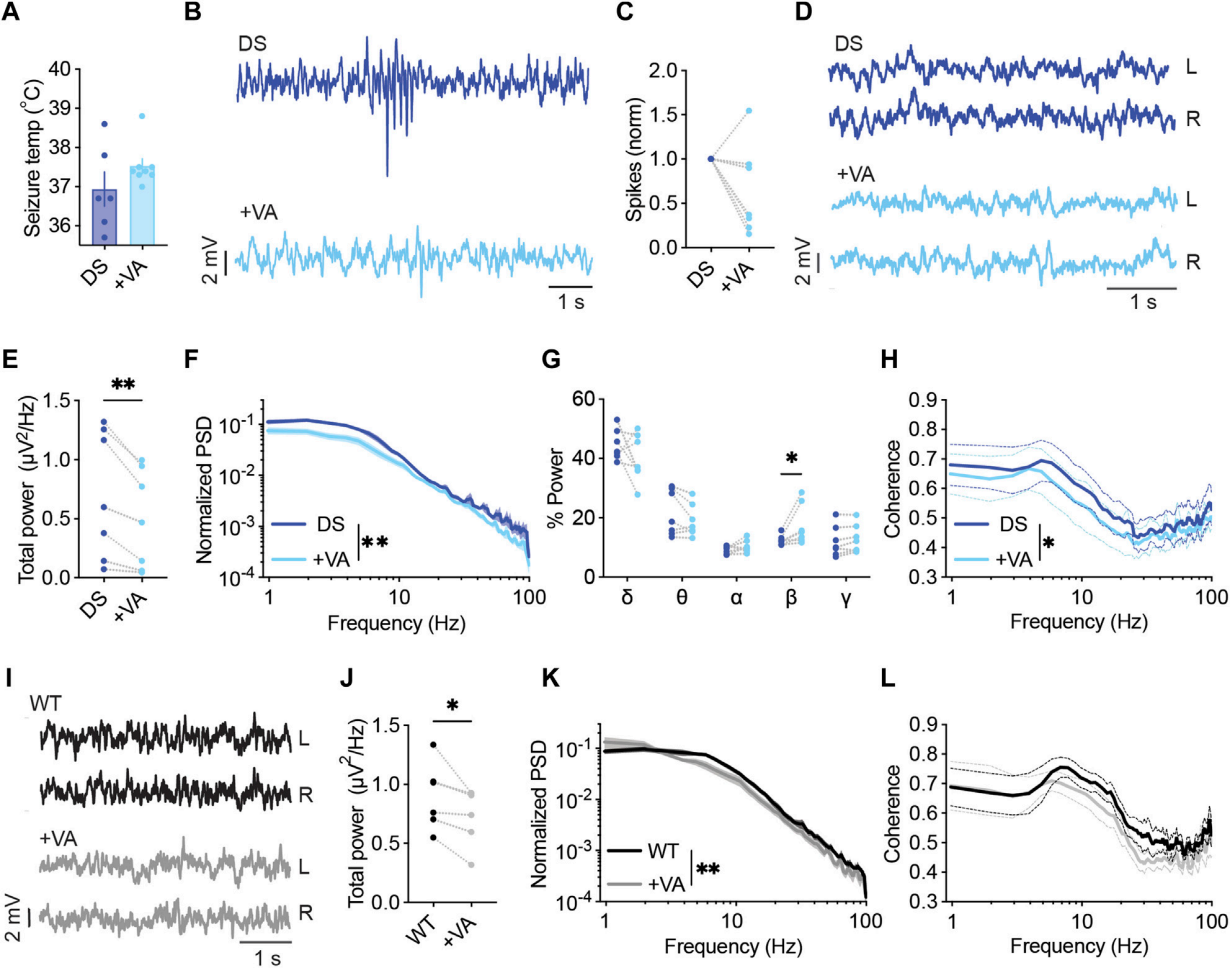
VA modulates the power and spectral properties of background ECoG. **(A)** Thermally induced seizures in mice treated with vehicle or VA (300 mg/kg). DS mice injected with saline as the vehicle, n =6; DS mice injected with VA, n = 8. **(B)** Representative traces of ECoGs from DS mice depicting epileptic activity before and after VA administration. **(C)** The change in interictal spike frequency. **(D)** Representative traces of background activity. **(E)** The effect of VA on total power. **(F)** ECoG power density profiles normalized to the total power prior to VA administration. **(G)** Relative power in each frequency band, before and after VA. **(H)** Interhemispheric coherence plotted over the 1–100 Hz spectrum. DS, n = 7. **(I)** Representative traces of background ECoGs from WT mice before and after VA administration. **(J)** The effect of VA on total power. **(K)** ECoG power density profiles normalized to the total power prior to drug administration. **(L)** Interhemispheric coherence plotted over the 1–100 Hz spectrum. WT, n = 5. *p < 0.05, **p < 0.01.

First, we tested the effect of VA on the threshold of heat-induced seizures. As shown in Figure 2A, mice treated with VA (300 mg/kg) tended to convulse at higher temperatures, but this difference did not reach statistical significance. Previous results reported similar observations using the same mouse model (*Scn1a* ^A1783V^ on the pure C57BL/6J background, (Pernici et al., 2021)). Conversely, others that used DS mice with a mixed genetic background demonstrated a significantly increased seizure threshold (Hawkins et al., 2017; Ho et al., 2021). Therefore, we repeated these experiments in *Scn1a* ^A1783V^ mice on a mixed C57BL/6J:129x1/SvJ background. VA significantly reduced the susceptibility to thermal seizures in these mice, increasing the average threshold temperature by 1.69°C ± 0.72°C (Supplementary Figure S2). C57BL/6J mice carry a variant of the *Gabra2* gene that results in reduced expression of the encoded GABA_A_ α2 subunits. This gene was shown to be a genetic modifier in Dravet (Hawkins et al., 2021). As VA also acts *via* the potentiation of GABA_A,_ this genetic variant in C57BL/6J may be related to the reduced effect of VA on heat-induced seizures. Thus, genetic background affects the ability of VA to protect from febrile seizures. However, as DS mice on a mixed background were reported before to have milder epileptic phenotypes (Yu et al., 2006; Mistry et al., 2014; Rubinstein et al., 2015), the severely affected DS mice on the pure C57BL/6J background were used for the rest of the study.

In a different cohort of mice on the C57BL/6J background, we tested the acute effect of VA on the frequency of interictal spikes and spectral properties of background ECoG. VA had a variable impact on spike frequency, reducing the frequency by over 50% in 57% of the tested mice, with a smaller effect in 28% of the mice, and increased spike frequency in one mouse (Figures 2B, C). Focusing on quantitative analyses of background activity, VA reduced the total power spectral density (PSD) in all the tested DS mice (Figures 2D–F), in agreement with previous reports in humans (Clemens et al., 2006; Moon et al., 2022). When inspecting the relative power of each frequency band, we observed an increase in the contribution of the beta band (Figure 2G). Despite the global effect on ECoG power, VA did not affect the coherence patterns in DS mice in any specific band. Yet, a slight overall reduction in phase coherence was observed (Figure 2H). The acute effect of VA was not specific to DS mice, and the reduction in total power was also seen in WT mice (Figure 2I–L, Supplementary Figure S3A). Together, although VA had variable effects on the frequency of interictal spikes in DS mice, it reduced the power and modulated the spectral properties of background ECoG activity.

**FIGURE 3:**
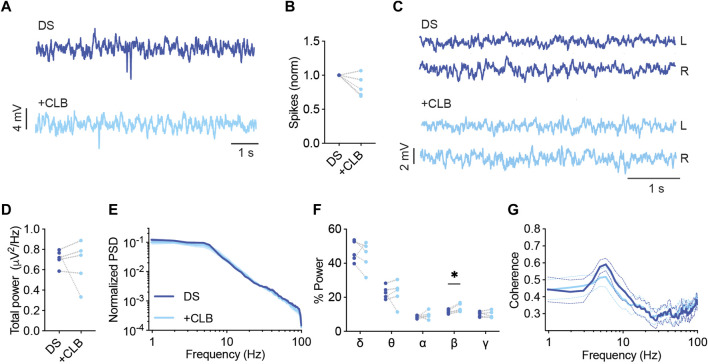
CLB treatment modulates the spectral properties of background ECoG in DS mice. **(A)** Representative traces of epileptic activity before and after acute administration of CLB (10 mg/kg) **(B)**. The change in the frequency of interictal spikes. **(C)** Representative examples of background activity. **(D)** The effect on total power. **(E)** ECoG power density profiles normalized to the total power prior to drug administration. **(F)** The relative power in each frequency band. **(G)** Interhemispheric coherence plotted over the 1–100 Hz spectrum. DS, n = 5. * *p* < 0.05.

Clobazam (CLB), frequently in combination with stiripentol (STP), is another commonly used standard treatment in Dravet (Strzelczyk and Schubert-Bast, 2022; Wirrell et al., 2022). As a monotherapy, clobazam was reported to be effective in 25%–50% of patients (Shi et al., 2016), while the addition of STP increased responsiveness. CLB is a long-acting 1,5-benzodiazepine that acts as a positive allosteric modulator of ionotropic GABA_A_ receptors (Hammer et al., 2015). CLB as monotherapy in mice was shown to protect from thermally induced seizures (Hawkins et al., 2017; Pernici et al., 2021). Here, CLB (10 mg/kg) reduced the frequency of interictal spikes in 80% of the mice (Figures 3A, B), had no effect on the total power or coherence (Figures 3C–E, G), but resulted in an increased contribution of the beta frequency band (Figure 3F).

STP is often administered as an adjunct drug. It enhances the effective concentration of CLB by inhibiting CYP450 isoenzymes. In addition, STP also directly modulates inhibitory GABAergic transmission *via* allosteric modulation of the GABA_A_ receptors, which enhances the positive effect of CLB on these channels. Moreover, STP also inhibits voltage-gated T-type calcium channels and lactate dehydrogenase (LDH), which was shown to suppress seizures (Fisher, 2011).

**FIGURE 4:**
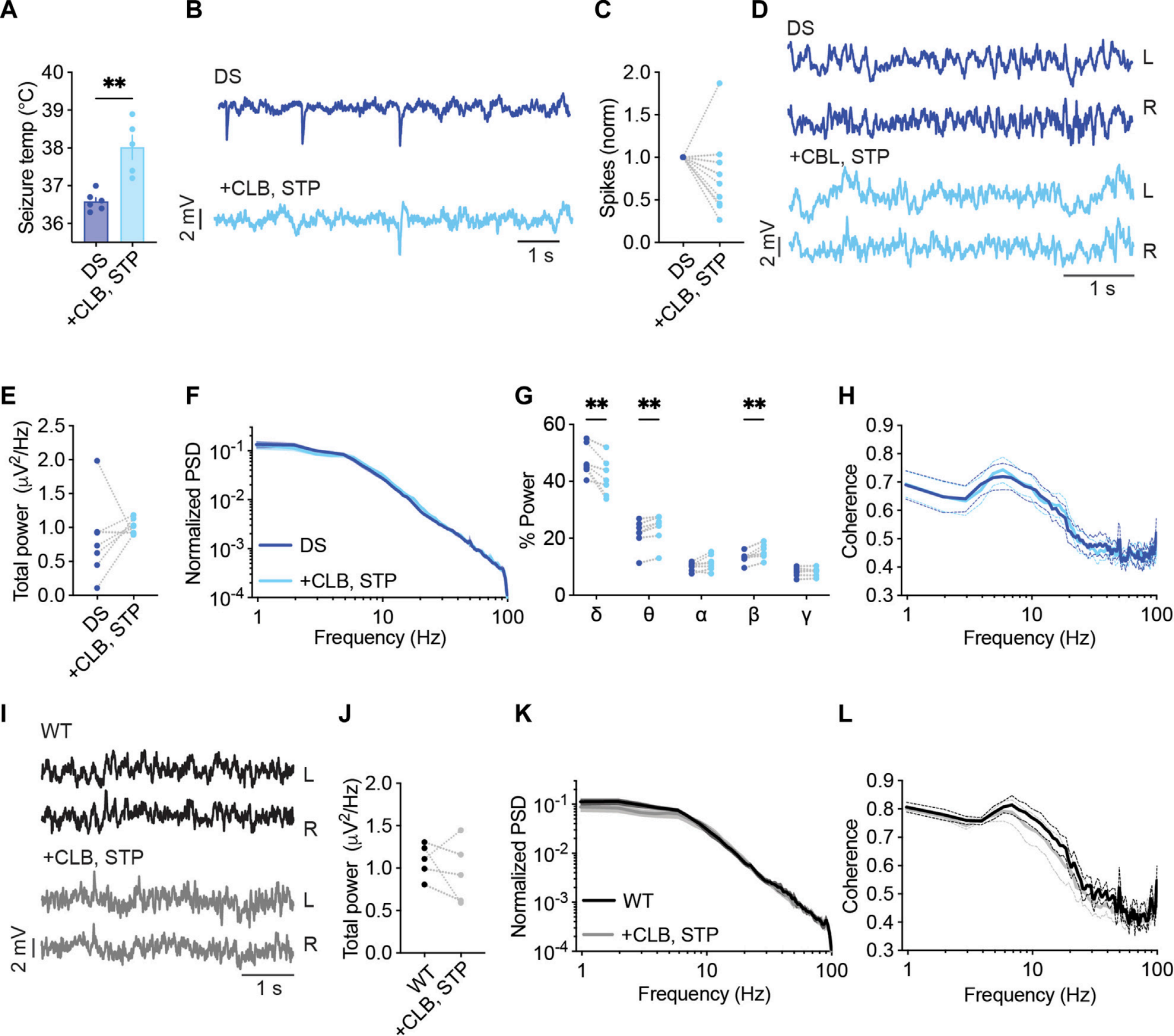
CLB + STP treatment modulates the spectral properties of background ECoG in DS mice. **(A)** CLB + STP (5,100 mg/kg, respectively) protected against hyperthermia-induced seizures in DS mice. DS mice injected with sesame oil as vehicle, n = 6; DS mice injected with CLB + STP, n = 5. **(B)** Representative traces of epileptic activity before and after acute administration of CLB + STP. **(C)** The change in the frequency of interictal spikes. **(D)** Representative examples of background activity. **(E)** The effect on total power. **(F)** ECoG power density profiles normalized to the total power prior to drug administration. **(G)** The relative power in each frequency band. **(H)** Interhemispheric coherence plotted over the 1–100 Hz spectrum. DS, n = 7. **(I)** Representative traces of background ECoG from WT mice before and after drug administration. **(J)** The effect of CLB + STP on total power. **(K)** ECoG power density profiles normalized to the power prior to drug administration. **(L)** Interhemispheric coherence plotted over the 1–100 Hz spectrum. WT, n = 5. ** *p* < 0.01.

Acute administration of CLB + STP (5 mg/kg, 100 mg/kg) significantly reduced the susceptibility to thermally induced seizures, elevating the average temperature of convulsive seizures by ∼ 1.4°C (Figure 4A). The effect of this drug combination was further tested using ECoG recordings. The frequency of interictal spikes decreased by over 50% in 33% of the mice, and by 20%–50% in another 33%, while no change or an increase in spike frequency was observed in the rest of the mice (Figures 4B, C). Moreover, we did not observe a significant effect on the total power of background activity in DS mice (Figures 4D–F). Nevertheless, acute administration of CLB + STP altered the relative power of the delta, theta, and beta frequency bands, reducing the delta contribution and increasing that of theta and beta (Figure 4G). The interhemispheric coherence did not change after CLB + STP acute administration (Figure 4H).

The effect of CLB + STP on WT mice was similar to that of DS mice, with no effect on the total power or coherence (Figures 4I–L), but with an enhancement of the relative power of the beta frequency band (Supplementary Figure S3B). Together, CLB + STP reduced the susceptibility to thermally induced seizures, reduced the frequency of interictal spikes in most of the tested mice, and modulated the spectral properties of background ECoG activity.

STP as monotherapy was shown to reduce seizure burden in patients (Hawkins et al., 2017). Neverthelss, it is usually administered as adjunct therapy (Wirrell et al., 2022), and was not shown to be protective against thermally induced seizures in mice (Hawkins et al., 2017; Pernici et al., 2021). With its direct effect on GABAergic transmission (Fisher, 2011), we wondered if it would also affect the spectral properties of background ECoG activity.

**FIGURE 5:**
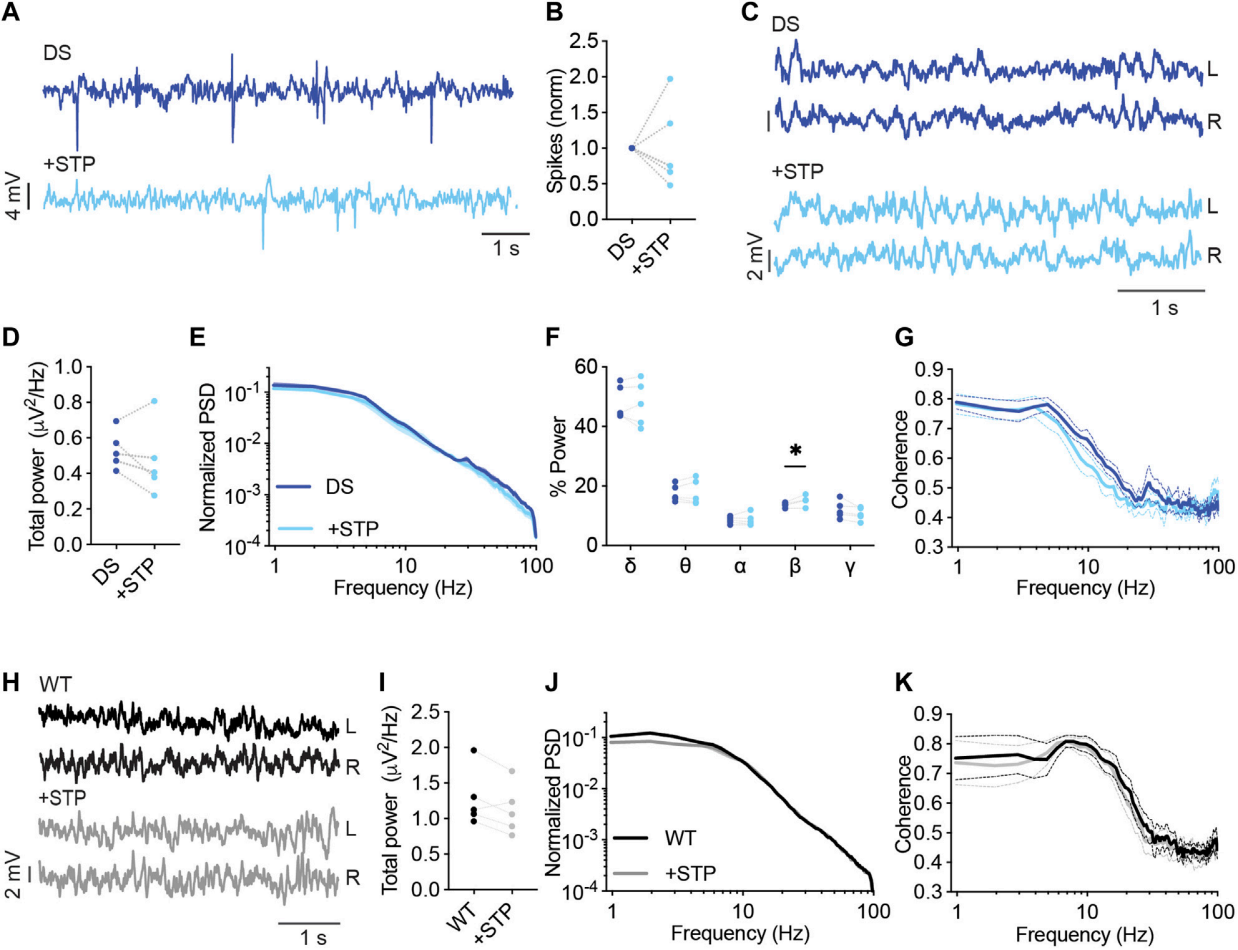
Analysis of background EcoG activity in response to STP administration. **(A)** Representative traces of epileptic activity in DS mice prior to and post, acute administration of STP (150 mg/kg). **(B)** The effect of STP on interictal spike frequency. **(C)** Representative traces of background ECoG in DS mice before and after STP administration. **(D)** The effect on total power. **(E)** ECoG power density profiles normalized to the absolute total power prior to drug administration. **(F)** The relative power in each frequency band. **(G)** Interhemispheric coherence plotted over the 1–100 Hz spectrum. DS, n = 5. **(H)** Representative traces of background ECoG from WT mice, before and after drug administration. **(I)** The effect of STP on total power. **(J)** ECoG power density profiles normalized to the absolute power prior to drug administration. **(K)** Interhemispheric coherence plotted over the 1–100 Hz spectrum. WT, n = 5. * *p* < 0.05.

STP (150 mg/kg) had variable effects on the frequency of interictal spikes, reducing their frequency by 25%–53% in three mice and increasing their frequency by 35%–90% in two other DS mice (Figures 5A, B). Moreover, while no significant effects were observed on the total power or coherence (Figures 5C–E, G), STP increased the contribution of the beta frequency band (Figure 5F). Together, Dravet-prescribed drugs, VA, CLB, CLB + STP, or STP alone, demonstrated variable, subject-dependent changes in the frequency of interictal epileptic spikes but with significant modulation of the spectral properties of background activity, and a common increase in the contribution of the beta frequency band.

### Contraindicated sodium channel blockers do not modulate the spectral parameters of background activity in DS mice

ASMs that act mainly via inhibition of voltage-gated sodium channels are contraindicated in Dravet. These drugs were shown to aggravate the epileptic phenotypes and adversely affect the cognitive outcome (de Lange et al., 2018; Wirrell et al., 2022). Carbamazepine (CBZ) mainly targets voltage-gated sodium channels. It has an increased affinity to the inactivated state, resulting in a voltage or frequency-dependent inhibition of these channels. However, CBZ was also demonstrated to inhibit the N-type and L-type voltage-gated calcium channels and increase the secretion of serotonin (Ambrósio et al., 2002).

**FIGURE 6:**
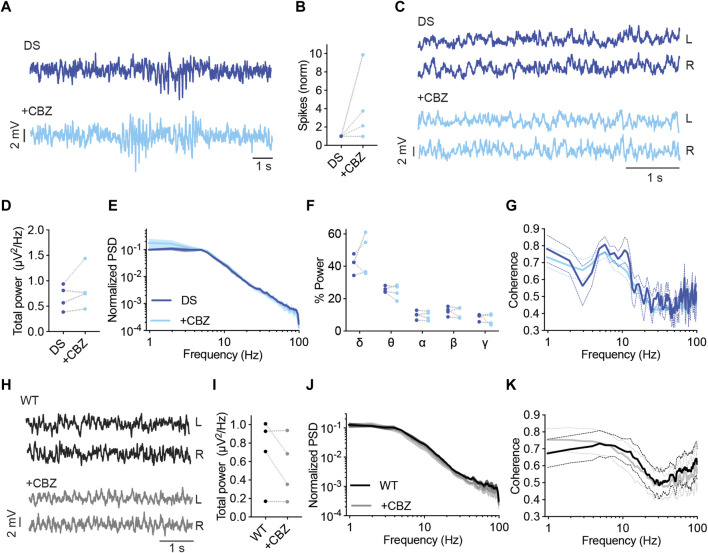
CBZ increased the frequency of interictal spikes in most DS mice. **(A)** Representative traces of epileptic activity in DS mice before and after acute administration of CBZ (20 mg/kg). **(B)** The effect of CBZ on interictal spike frequency. **(C)** Representative traces of background ECoG in DS mice before and after treatment with CBZ. **(D)** The effect on total power. **(E)** ECoG power density profiles normalized to the absolute total power prior to drug administration. **(F)** The relative power in each frequency band. **(G)** Interhemispheric coherence plotted over the 1–100 Hz spectrum. DS, n = 4. **(H)** Representative traces of background ECoG from WT mice, before and after administration of CBZ. **(I)** The effect of CBZ on total power. **(J)** ECoG power density profiles normalized to the absolute power prior to drug administration. **(K)** Interhemispheric coherence plotted over the 1–100 Hz spectrum. WT, n = 4.

CBZ was reported to exacerbate the susceptibility to thermally induced seizures in DS mice (Pernici et al., 2021), but its effect on ECoG and background activity was not reported. Here, among the mice treated with CBZ (20 mg/kg), three out of four mice demonstrated an increased frequency of epileptic spikes by 2-9 folds, and a marginal (3%) decrease in spike frequency was seen in the fourth mouse (Figures 6A, B). Background spectral properties analysis showed that CBZ did not modify the power, spectral profiles, or coherence (Figures 6C–G). Similarly, in WT mice, CBZ did not modify the properties of background oscillations (Figures 6H–K).

**FIGURE 7:**
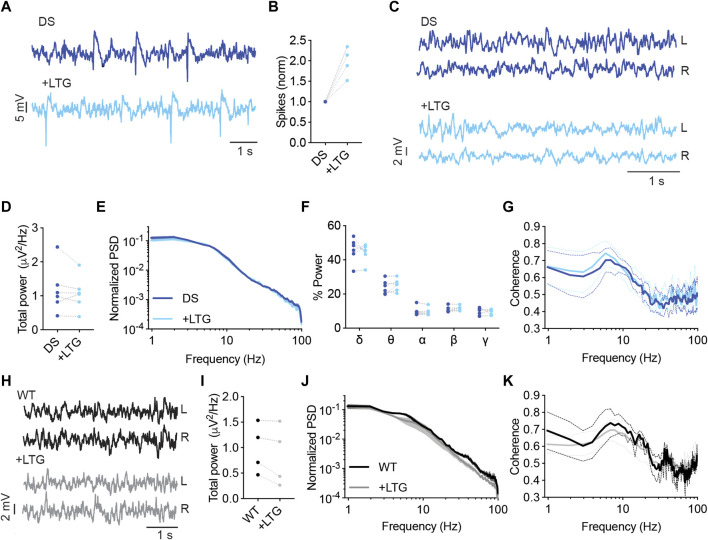
LTG increased the frequency of interictal spikes in DS mice. **(A)** Representative traces of epileptic activity in DS mice before and after acute administration of LTG (10 mg/kg). **(B)** The effect of LTG on interictal spike frequency. **(C)** Representative traces of background ECoG in DS mice before and after treatment with LTG. **(D)** The effect on total power. **(E)** ECoG power density profiles normalized to the absolute total power prior to drug administration. **(F)** The relative power in each frequency band. **(G)** Interhemispheric coherence plotted over the 1–100 Hz spectrum. DS, n =6. **(H)** Representative traces of background ECoG from WT mice, before and after administration of LTG. **(I)** The effect of LTG on total power. **(J)** ECoG power density profiles normalized to the absolute power prior to drug administration. **(K)** Interhemispheric coherence plotted over the 1–100 Hz spectrum. WT, n = 4.

Lamotrigine (LTG) is another voltage-gated sodium channel blocker avoided in Dravet (Wirrell et al., 2022). Additional mechanisms of action include inhibition of voltage-gated calcium channels and reduced presynaptic glutamate release (Prabhavalkar et al., 2015). In DS mice, acute administration of LTG (10 mg/kg) resulted in increased interictal spike frequency (Figures 7A, B). Moreover, LTG did not affect the power or spectral properties of background activity (Figures 7C–G). Together, acute administration of either CBZ or LTG increased the frequency of interictal spikes in DS mice without affecting the properties of background oscillations.

### Correlation between interictal spike frequency, ECoG power, and spectral properties

We wondered if comprehensive analyses of the various drug-induced changes would highlight parameters that can differentiate between Dravet-prescribed and contraindicated drugs and correlate with the effect on the epileptic phenotypes as measured here by the frequency of interictal spikes.

**FIGURE 8:**
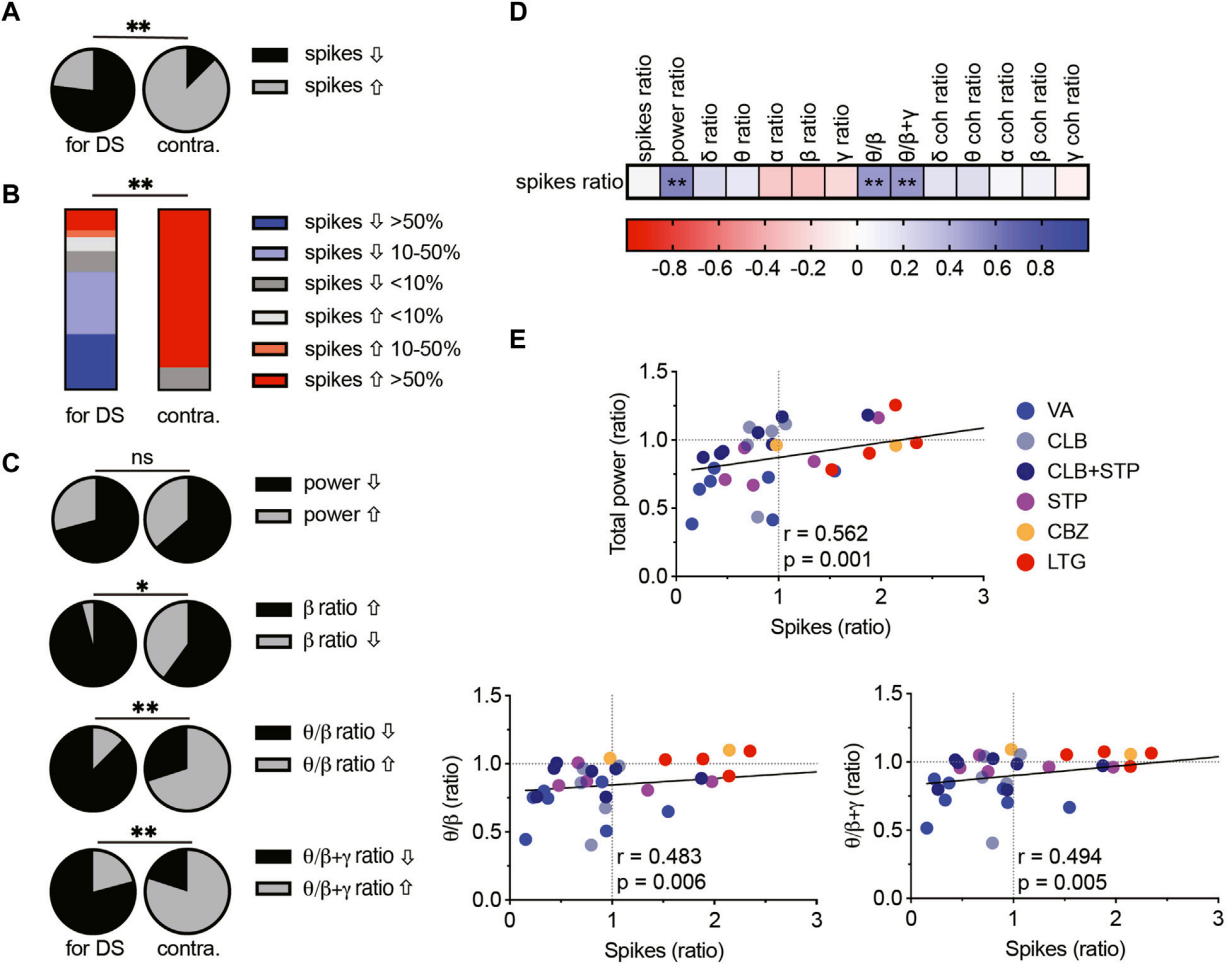
Correlations between spike frequency and spectral parameters in DS mice. **(A)** The proportion of mice with increased or decreased spike frequency among prescribed (left, VA, CLB, CLB + STP, STP) and contraindicated (right, CBZ, LTG) ASMs in DS mice. **(B)** Percentages of mice and their change in spike frequency following treatment with prescribed (left) and contraindicated (right) ASMs. **(C)** The proportion of DS mice, treated with prescribed (left) and contraindicated (right) ASMs with increased or decreased total power, beta contribution, theta/beta relative power ratios, or theta/(beta + gamma) ratios. **(D)** Correlation matrix (Spearman correlation) between the change in the frequency of interictal spikes, background spectral parameters, and the coherence (coh). The change was calculated as the ratio between a specific parameter after ASM administration divided by the same parameter before ASMs. Full correlation analysis is presented in Supplementary Fig. 5. A full description of the correlation coefficients and statistical significance are presented in Supplementary Table 1. **(E)** Correlations between the change in spike frequency and the total power (top), the theta/beta ratio (bottom, left), and the theta/(beta + gamma) ratio (bottom, right). The text indicates the Spearman correlation coefficient and statistical significance. The solid black line depicts the fit. * *p* < 0.05, ** *p* < 0.01.

First, we quantified the overall effect of ASMs on spike frequency. VA, CLB, or CLB + STP reduced the spike frequency in ∼80% of the mice (Figures 2–4), while STP lowered their frequency in 60% of the tested mice (Figure 5). Overall, Dravet-recommended ASMs reduced spike frequency by over 50% in 30% of the mice and by over 10% in 66% of the mice. Conversely, contraindicated drugs increased the spike frequency by more than 50% in 87.5% of the tested mice (Figures 8A, B).

Next, we examined the relationship between background spectral changes and the effect on interictal spikes (see Supplementary Figure S5 for the complete correlation analyses of spectral changes). Focusing on the total power shift, we did not see a statistical difference between the effect of Dravet-recommended and contraindicated drugs (Figure 8C). However, interestingly, the level of total power reduction correlated with a decrease in spike frequency (Figures 8D, E). This correlation was also significant for the cohort of mice treated with CLB + STP (Supplementary Figure S4). Examination of the spectral power redistribution showed an increase in the relative beta contribution among the majority of mice treated with Dravet-prescribed ASMs and a less profound change after CBZ and LTG administration (Figure 8C). Nevertheless, the relative drug-induced change in the beta band contribution did not correlate with the reduction in spike frequency (Figure 8D). Thus, we wondered if power redistribution across multiple bands would correlate with the effect on interictal spikes. The ratio between theta and beta has been extensively investigated in relation to attention processes and is FDA-approved for clinical confirmation in attention deficit disorder (Clarke et al., 2020). Interestingly, a reduction in this ratio correlated with a reduction in spike frequency. A similar trend was observed when we calculated the ratio of theta to beta and gamma combined (Figures 8C–E). Together, examination of multidimensional readouts for the effect of acute antiseizure treatment on background activity and epileptic interictal spikes revealed a correlation between the frequency of interictal spikes, background power, and the relative contribution of theta, beta and gamma frequency bands.

## Discussion

Dravet syndrome is characterized by multiple neuronal and circuitry changes (Mantegazza and Broccoli, 2019). Here, by examining background activity in DS mice and their WT littermates, we show attenuated power of background activity in DS mice, similar to previous reports (Fadila et al., 2020; Gerbatin et al., 2022), and add a reduction in interhemispheric phase coherence (Figure 1). Although epileptic activity is characterized by hypersynchronous firing (Staba and Worrell, 2014) (Supplementary Figure S1), seizure onset was shown to be associated with a reduced synchronization (Schindler et al., 2007), and transition to thermally induced seizures in DS mice was associated with decreased synchronization of excitatory and inhibitory neurons (Tran et al., 2020). In Alzheimer’s disease (AD) patients, reduced neural synchrony was associated with AD-related cognitive deficits (Stam et al., 2007). Therefore, it is possible that decreased background synchronization is also related to developmental delay and cognitive deficits in Dravet.

Interestingly, none of the tested ASMs corrected the attenuated power or coherence (Figures 1–5), and a reduction in total power correlated with a decrease in the frequency of interictal spikes (Figure 8). However, as low power was suggested as a risk factor for premature mortality in DS mice (Fadila et al., 2020; Gerbatin et al., 2022), an ideal treatment for Dravet can be expected to amend these parameters rather than further decrease the power. Thus, it may be that the observed association between reduced power and reduction in spike frequency provides an electrographic measurement for the partial ability of ASMs to correct Dravet, stressing the need for developing novel treatment options.

Examination of the power redistribution following acute drug administration demonstrated that Dravet-prescribed ASMs modulate the spectral properties of background activity, mainly by affecting the contribution of the beta frequency band. In accordance, increased beta power was observed in patients on benzodiazepines or barbiturates (Armstrong et al., 2022), and acute administration of VA (Clemens et al., 2006) or CLB (Gerhards, 1978) was shown to increase the contribution of beta. Moreover, increased relative beta power was observed in medicated Dravet patients at the age of 1–2 years compared to unmedicated age match controls (Holmes et al., 2012). Of note, this age represents the onset of spontaneous seizures, which is somewhat parallel to the developmental stage of the mice used here. Furthermore, an increase in beta contribution was also suggested to predict the positive effect of cannabidiol as an add-on therapy in patients treated with CLB (Armstrong et al., 2022). Despite that, in our cohort, the relative change in beta band contribution alone did not correlate with the reduction in spike frequency (Figures 8C, D). However, a broader examination of the power redistribution between theta, beta, and gamma bands, demonstrated a correlation with a reduction in spike frequency (Figures 8C–E). Interestingly, the ratio between theta and beta was suggested to be a reliable electrographic measurement that reflects cognitive processing and was therefore indicated as a biomarker in patients with attention deficit disorder (Clarke et al., 2020). Thus, we propose that, following additional studies, examining these background activity changes subsequently to the administration of ASMs, may be an additional tool to evaluate the therapeutic potential of drug treatment also in Dravet.

One limitation of the current study is that the effect on epilepsy was quantified as the change in the frequency of the interictal spikes using short-term ECoG recordings, due to challenges with prolonged recordings in immature mice. Conversely, in clinical settings, drug treatment is focused on seizure reduction and prevention of status epilepticus over a long time. Nevertheless, despite the apparent difference in readout (interictal spikes vs. spontaneous seizures) and drug regimen (acute vs. chronic), the overall response levels reported here resembled clinical reports (Inoue et al., 2009; Shi et al., 2016; Hawkins et al., 2017). Specifically, patients show 50%–80% responsiveness levels, compared to a reduction in spike frequency in ∼80% of the tested mice (Figures 2–5, 8). In contrast, drugs known to aggravate Dravet epileptic phenotypes increased the frequency of spikes (Figures 6–8).

Interestingly, even though we used DS mice of the same age, *Scn1a* mutation (A1783V), and C57BL/6J genetic background, we still observed variable responses that did not correlate with age, weight, litter size or the sex of the mice tested (Supplementary Table S2). Thus, it is possible that distinct individual epileptogenic processes or various homeostatic changes contribute to personalized drug responses, even in animal models.

Together, this study focused on measurements of background properties and interictal spikes in mice. Many of these parameters, including the lower interhemispheric coherence compared to WT mice, measurements of the absolute power that are sensitive to the equipment used, recording quality, and the developmental stage, are mainly suitable for pre-clinical studies. Nevertheless, following additional validation, some parameters, such as alteration in the spectral properties or the correlation between the ratio of theta to beta and gamma, can potentially be adopted for clinical use (Figure 8).

In conclusion, we describe the acute effects of ASMs on ECoG spectral properties in DS mice. Our analyses demonstrate a distinct impact of Dravet-prescribed drugs on ECoG spectral properties and interictal spikes frequency, suggesting that such measurements can potentially be used as additional informative readouts for the therapeutic benefit of drug therapy in Dravet.

## Data Availability

The raw data supporting the conclusion of this article will be made available by the authors, without undue reservation.
