# Supplementary material for: Acute effect of antiseizure drugs on background oscillations in Scn1a A1783V Dravet syndrome mouse model
Source: Front Pharmacol. 2023 Mar 20;14:1118216. doi: 10.3389/fphar.2023.1118216 (PMC10067575; doi:10.3389/fphar.2023.1118216)
Supplement: Supplementary file 1 [file Image1.pdf]

Supplementary material

Acute effect of antiseizure drugs on background oscillations in *Scn1a*^{A1783V} Dravet syndrome mouse model

Shir Quinn, Marina Brusel, Mor Ovadia, Moran Rubinstein

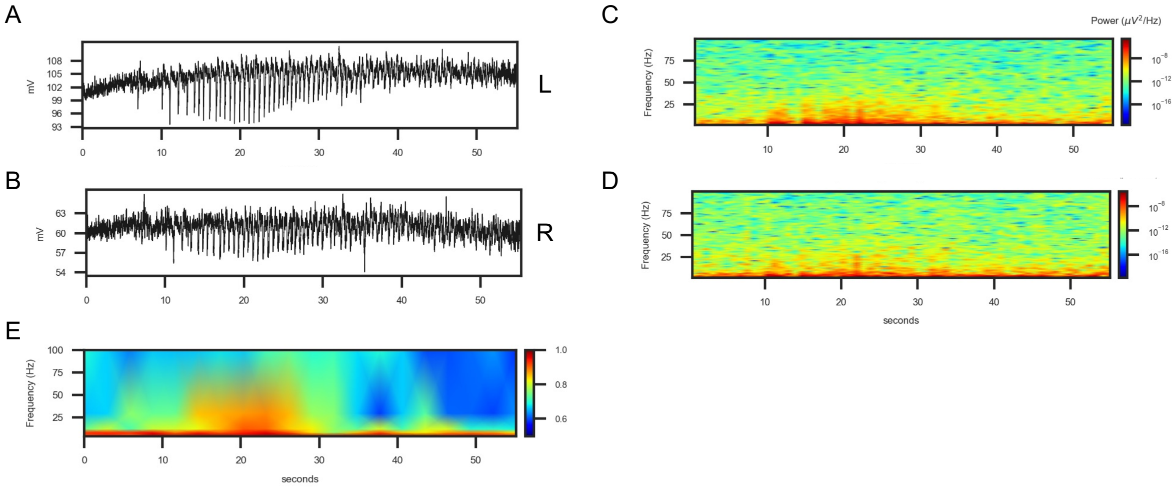

Supplementary Fig. 1. The power and coherence during spontaneous seizure activity in a Dravet mouse. Depicted are the raw voltage trace in the left (A) and right (B) cortical electrodes, the power in each channel (C, D), and the coherence (E).

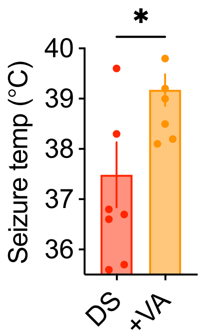

Supplementary Fig. 2. Valproic acid (300 mg/kg) decreased the susceptibility of thermally induced seizures in *Scn1a*$^{A1783V/WT}$ mice on a mixed background (50:50 C57BL/6J:129x1/SvJ).

The mice were generated by crossing *Scn1a*$^{A1783V/WT}$ males (pure C57BL/6J) with WT females (129x1/SvJ). DS injected with saline n=7; DS injected with VA n=6.

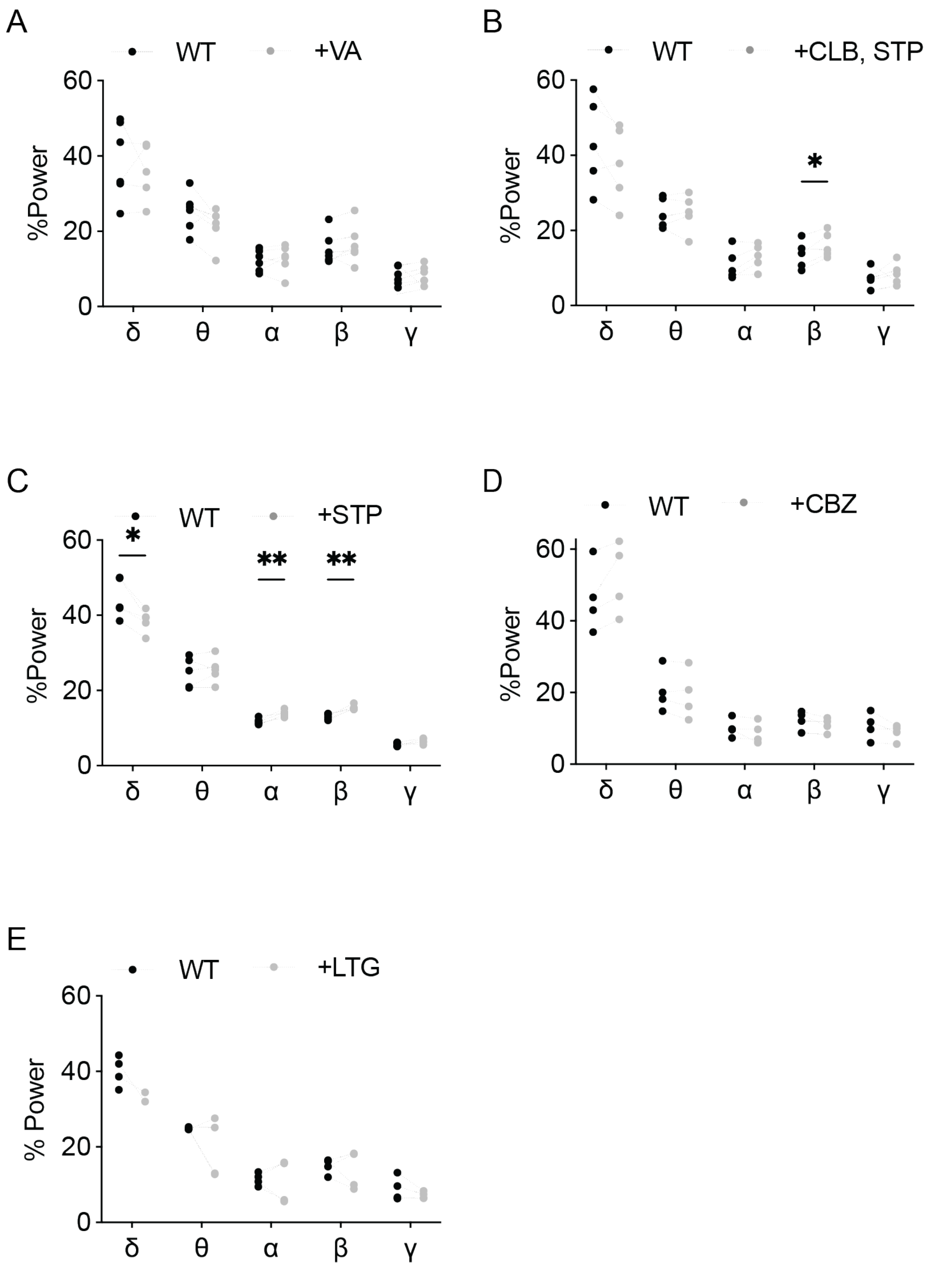

Supplementary Fig. 3. The effect of ASMs on spectral properties of WT mice.

(A) Relative power in each frequency band, before and after administration of VA, related to Figure 2I-K. n = 6. **(B)** Relative power in each frequency band, before and after administration of CLB+STP, related to Figure 4I-K. n = 5. **(C)** Relative power in each frequency band, before and after administration of STP, related to Figure 5H-KJ. n = 5. **(D)** Relative power in each frequency band, before and after administration of CBZ, related to Figure 6H-J. n = 4. **(E)** Relative power in each frequency band, before and after administration of LTG, related to Figure 7H-J. n = 4. *$p < 0.05$, **$p < 0.01$.

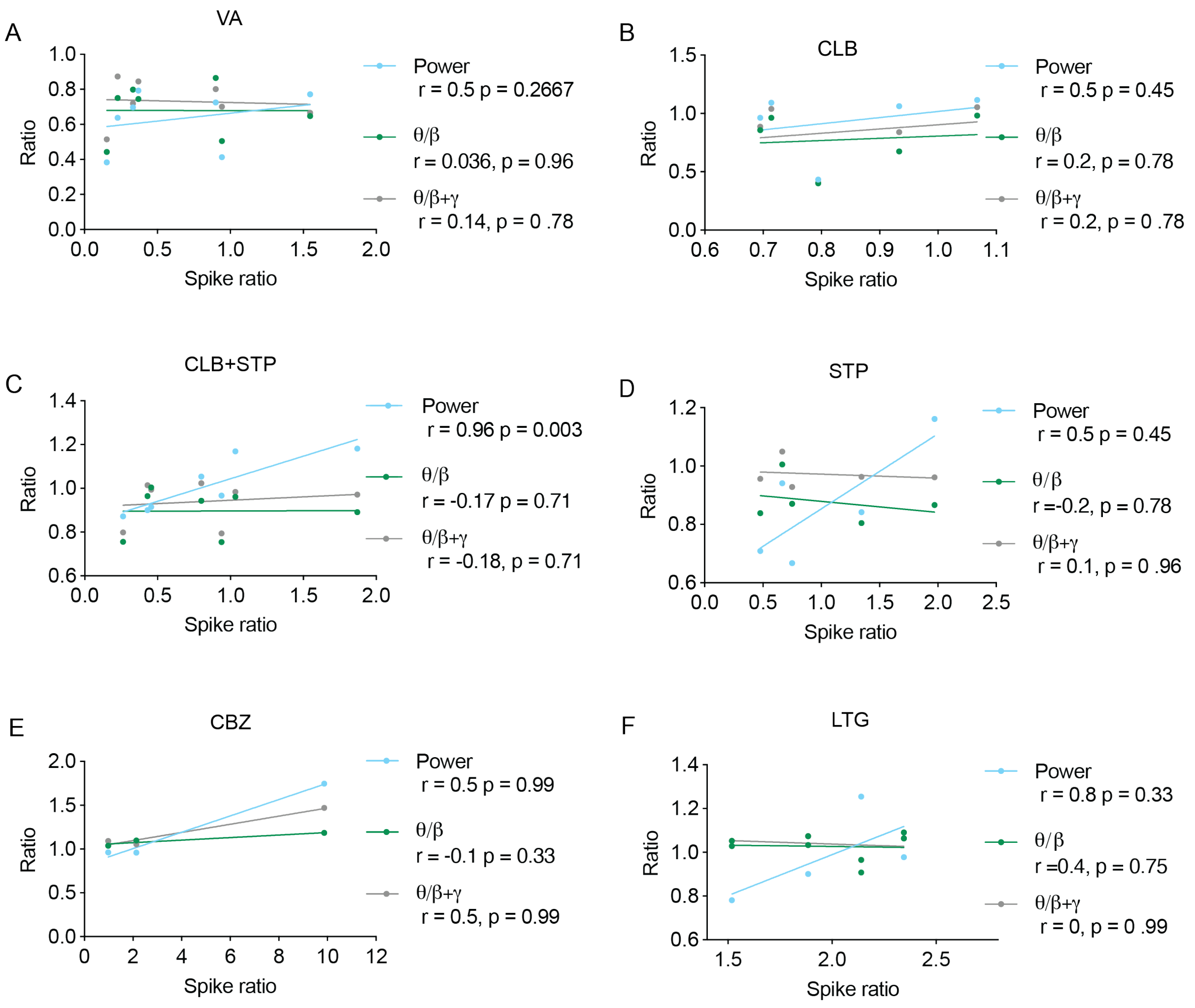

Supplementary Fig. 4. Correlations between spike frequency and spectral parameters in DS mice.

Correlation between the change in total power, theta /beta ratios, and theta /(beta + gamma) ratios in DS mice treated with **(A)** VA, n = 7. **(B)** CLB, n=5; **(C)** CLB+STP, n = 9. **(D)** STP, n = 5. **(E)** CBZ, n = 3. **(F)** LTG, n = 4. The correlation coefficients (Spearman, r) and p-values (p) are depicted. The solid line is the result of simple linear regression and is depicted for illustration purposes. Related to Figure 6E.

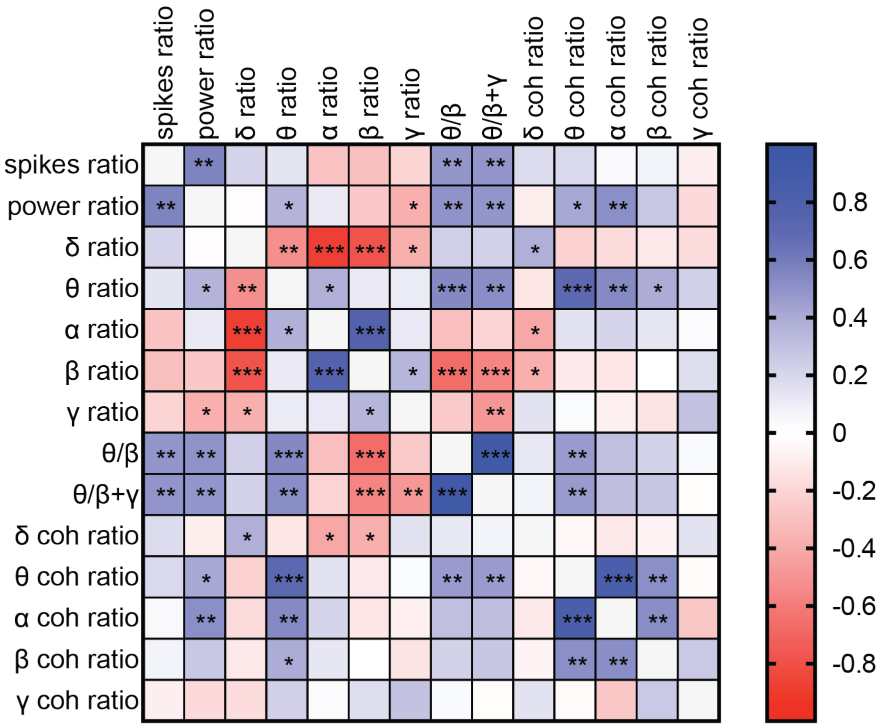

Supplementary Fig. 5. Correlations between spike frequency and spectral parameters in DS mice. The full Correlation matrix (Spearman correlation) between the change in the frequency of interictal spikes, background spectral parameters and the coherence (coh). A full description of the correlation coefficients and statistical significance are presented in Supplementary Table 1.

Supplementary Table 1 **– Correlations between spike frequency and spectral parameters in DS mice**. Spearman correlation coefficient (A) and p values (B). Related to Figure 8 and Supplementary Figure 6

A. Spearman correlation coefficient

	spikes ratio	power ratio	δ ratio	θ ratio	α ratio	β ratio	γ ratio	θ/β	θ/β+γ	δ coh ratio	θ coh ratio	α coh ratio	β coh ratio	γ coh ratio
spikes ratio	1.0000	0.5617	0.2101	0.1310	-0.2867	-0.3020	-0.2109	0.4831	0.4940	0.1755	0.1915	0.0394	0.0719	-0.0923
power ratio	0.5617	1.0000	-0.0218	0.3613	0.1111	-0.2660	-0.3837	0.5016	0.4848	-0.0972	0.4194	0.5105	0.2702	-0.1806
δ ratio	0.2101	-0.0218	1.0000	-0.5236	-0.8772	-0.7873	-0.3751	0.2281	0.2196	0.3774	-0.2230	-0.1774	-0.1169	-0.1698
θ ratio	0.1310	0.3613	-0.5236	1.0000	0.3742	0.1123	0.1028	0.5404	0.5154	-0.1290	0.7060	0.5359	0.3992	0.2298
α ratio	-0.2867	0.1111	-0.8772	0.3742	1.0000	0.7598	0.1181	-0.3131	-0.2150	-0.4157	0.1468	0.2105	0.1286	0.0234
β ratio	-0.3020	-0.2660	-0.7873	0.1123	0.7598	1.0000	0.3476	-0.6743	-0.5664	-0.3831	-0.1109	-0.1282	-0.0004	0.1637
γ ratio	-0.2109	-0.3837	-0.3751	0.1028	0.1181	0.3476	1.0000	-0.2626	-0.4839	0.1431	0.0266	-0.0843	-0.1310	0.2903
θ/β	0.4831	0.5016	0.2281	0.5404	-0.3131	-0.6743	-0.2626	1.0000	0.9160	0.1246	0.4641	0.3097	0.2173	0.0335
θ/β+γ	0.4940	0.4848	0.2196	0.5154	-0.2150	-0.5664	-0.4839	0.9160	1.0000	0.0694	0.4665	0.3105	0.2718	-0.0294
δ coh ratio	0.1755	-0.0972	0.3774	-0.1290	-0.4157	-0.3831	0.1431	0.1246	0.0694	1.0000	-0.0524	-0.1153	-0.0661	0.1415
θ coh ratio	0.1915	0.4194	-0.2230	0.7060	0.1468	-0.1109	0.0266	0.4641	0.4665	-0.0524	1.0000	0.8222	0.5113	-0.0339
α coh ratio	0.0394	0.5105	-0.1774	0.5359	0.2105	-0.1282	-0.0843	0.3097	0.3105	-0.1153	0.8222	1.0000	0.5210	-0.2669
β coh ratio	0.0719	0.2702	-0.1169	0.3992	0.1286	-0.0004	-0.1310	0.2173	0.2718	-0.0661	0.5113	0.5210	1.0000	0.2629
γ coh ratio	-0.0923	-0.1806	-0.1698	0.2298	0.0234	0.1637	0.2903	0.0335	-0.0294	0.1415	-0.0339	-0.2669	0.2629	1.0000

B. P values

	spikes ratio	power ratio	δ ratio	θ ratio	α ratio	β ratio	γ ratio	θ/β	θ/β+γ	δ coh ratio	θ coh ratio	α coh ratio	β coh ratio	γ coh ratio
spikes ratio		0.0010	0.2567	0.4822	0.1179	0.0987	0.2548	0.0059	0.0047	0.3535	0.3106	0.8363	0.7059	0.6275
power ratio	0.0010		0.9024	0.0358	0.5317	0.1284	0.0251	0.0025	0.0037	0.6030	0.0189	0.0033	0.1416	0.3308
δ ratio	0.2567	0.9024		0.0015	0.0000	0.0000	0.0288	0.1944	0.2122	0.0363	0.2279	0.3397	0.5310	0.3613
θ ratio	0.4822	0.0358	0.0015		0.0293	0.5272	0.5628	0.0010	0.0018	0.4891	0.0000	0.0019	0.0261	0.2136
α ratio	0.1179	0.5317	0.0000	0.0293		0.0000	0.5059	0.0714	0.2221	0.0200	0.4308	0.2557	0.4904	0.9006
β ratio	0.0987	0.1284	0.0000	0.5272	0.0000		0.0440	0.0000	0.0005	0.0334	0.5526	0.4918	0.9983	0.3789
γ ratio	0.2548	0.0251	0.0288	0.5628	0.5059	0.0440		0.1334	0.0037	0.4424	0.8870	0.6522	0.4822	0.1131
θ/β	0.0059	0.0025	0.1944	0.0010	0.0714	0.0000	0.1334		0.0000	0.5042	0.0085	0.0900	0.2402	0.8581
θ/β+γ	0.0047	0.0037	0.2122	0.0018	0.2221	0.0005	0.0037	0.0000		0.7108	0.0082	0.0891	0.1391	0.8751
δ coh ratio	0.3535	0.6030	0.0363	0.4891	0.0200	0.0334	0.4424	0.5042	0.7108		0.7794	0.5367	0.7238	0.4476
θ coh ratio	0.3106	0.0189	0.2279	0.0000	0.4308	0.5526	0.8870	0.0085	0.0082	0.7794		0.0000	0.0033	0.8565
α coh ratio	0.8363	0.0033	0.3397	0.0019	0.2557	0.4918	0.6522	0.0900	0.0891	0.5367	0.0000		0.0027	0.1466
β coh ratio	0.7059	0.1416	0.5310	0.0261	0.4904	0.9983	0.4822	0.2402	0.1391	0.7238	0.0033	0.0027		0.1530
γ coh ratio	0.6275	0.3308	0.3613	0.2136	0.9006	0.3789	0.1131	0.8581	0.8751	0.4476	0.8565	0.1466	0.1530	

Supplementary Table 2 **– Correlations between spike frequency and age sex and weight of DS mice**.

A. Spearman correlation coefficient

	Date of birth	Age (day of recordings)	sex	litter size	Weight (day of surgery)	Weight (day of recoridng)	Change in spike
**Change in spike**	0.1927	-0.3520	-0.1363	-0.3006	-0.0434	0.0196	1.0000

B. P values

	Date of birth	Age (day of recordings)	sex	litter size	Weight (day of surgery)	Weight (day of recoridng)	Change in spike
**Change in spike**	0.4435	0.1520	0.5897	0.2256	0.8643	0.9384	
